# Integrating mental and behavioral health providers into VHA pain management teams and oncology clinics: assessment of early implementation from site visits

**DOI:** 10.3389/frhs.2026.1863653

**Published:** 2026-09-01

**Authors:** Molly Goodrich, Benjamin R. Szymanski, Brittany L. Cornwell, Kristen Perry, Katherine M. Dollar, Jennifer Patterson, John F. McCarthy

**Affiliations:** 1 Veterans Affairs (VA) Serious Mental Illness Treatment Resource and Evaluation Center, Office of Mental Health, Ann Arbor, MI, United States; 2 Center for Integrated Healthcare, Veterans Health Administration, Syracuse, NY, United States; 3 Office of Mental Health, Veterans Health Administration, Washington, DC, United States; 4 Department of Psychiatry, University of Michigan, Ann Arbor, MI, United States

**Keywords:** behavioral health, integrated care, oncology, pain, Veterans

## Abstract

**Introduction:**

To enhance mental health access and suicide prevention services for Veteran patients in Pain Management Teams and Oncology Clinics, the Veterans Health Administration (VHA) initiated the Mental and Behavioral Health Integration (MBHI) Demonstration Projects at 53 facilities in 2022. MBHI integrates colocated mental health providers into these settings to deliver brief mental/behavioral healthcare. Findings describe site visits assessing early implementation.

**Methods:**

Evaluators visited nine VHA facilities where MBHI providers were recruited and implementation was at early stages. Visits included program overviews, tours, and provider and site leadership interviews. Visits had consistent agendas and a framework of questions assessing fidelity to core components, site-specific modifications, and patients and conditions treated. Observations were content coded and organized thematically.

**Results and Discussion:**

MBHI services were available at each facility. Sites varied regarding clinic space for MBHI, brief treatment model use, and MBHI same-day access availability. All sites reported positive impacts for Veterans, clinical operations, and facilities. Implementation strengths and facilitators included local buy-in, MBHI provider integration into teams, utilizing partnerships and resources, MBHI provider experience and approach to care, and clinic workflow. Implementation challenges included space limitations, competing MBHI provider demands, productivity demands, uncertainty about intended patient populations, difficulty integrating MBHI staff, and hiring challenges and specialty clinic nascency. Visits identified positive impacts of MBHI, facilitators, and barriers to program implementation. Information gathered from site visits can inform VA policies and operations. Results continue to inform program modifications including enhancing role clarity, medical provider buy-in, and ongoing competency training.

## Introduction

Enhancing Veteran mental health access and suicide prevention are key priorities of the Veterans Health Administration (VHA), which is the largest integrated healthcare system in the United States (U.S.) (1). Through a collaborative effort between the Office of Mental Health (OMH), Office of Suicide Prevention (OSP), and Specialty Care Program Office to further expand and improve VHA's ability to reach Veterans at high risk of suicide, VHA initiated Demonstration Projects for Mental and Behavioral Health Integration (MBHI) into Pain Management Teams (PMTs) and Oncology Clinics in 2022 (2). The aim is to provide integrated mental and behavioral health services to Veteran patients seen by VHA PMTs and Oncology Clinics.

VHA has substantial experience developing and enacting integrated mental health services, including national VHA implementation of Primary Care Mental Health Integration (PCMHI) programs (3, 4). Drawing from this experience and with the understanding that suicide risk and mental health treatment needs are elevated among Veterans with new cancer diagnoses (5) and those with chronic pain (6), the Demonstration Projects were initiated in order to improve identification, treatment, and management of comorbid mental and behavioral health needs among VHA Pain Clinic and Oncology Clinic patients. The program's Request for Applications (RFA) was issued in March 2022 and included the following objectives: (1) advancing the capacity to complete suicide risk assessments and provide same-day access for mental and behavioral health needs in these settings; (2) increasing identification of co-occurring mental health needs for these patients; (3) improving access for and engagement with Veterans within medical populations (including those at higher risk for suicide) who are experiencing a broad range of mental health and health behavior concerns; and (4) increasing access to and provision of brief mental and behavioral interventions to address these needs (including referral to and coordination with specialty mental health).

Facilities that received Demonstration Projects funding did so through a competitive application process in response to the RFA. Applications were reviewed by OMH Integrated Services leadership. Funding decisions considered factors including the strength of the proposed integrated care model, existing infrastructure and leadership support, reported need for embedded mental health and integrated care, and geographic distribution across VISNs.

The RFA outlined expectations for the MBHI provider role such as delivery of same-day services, including brief mental health treatment, behavioral health treatment and intervention, support for Veterans’ engagement and coordination with specialty mental health providers as needed, and consultation supports for clinic medical care providers. Sites could apply for up to 2 FTEs per program, which could include a combination of mental health providers from different backgrounds (e.g., psychologists, licensed clinical social workers, psychiatrists). Funding for a limited number of 0.25−0.50 FTE providers (e.g., to provide medication services) was also considered. Consistent with the MBHI Practice Management Guidance, MBHI providers were expected to serve as integrated care generalists, with specialty-specific knowledge, skills, and abilities that could be acquired or expanded post-hire.

More detailed information regarding implementation and practice management expectations was provided to the field via a Program Parameters worksheet (2), which identified eight core categories: population; screening, referral to care, and follow-up; treatment of common conditions; health behavior knowledge and skills; patient health and well-being; interdisciplinary teamwork; systematic quality improvement; and sustainment. Within these categories, specific parameters include: daily same-day open access as a critical program component; initial assessments of 30 minutes or less focused on the primary reason for referral and its functional impact; brief, time-limited follow-up interventions (typically ≤30 minutes and 1−6 sessions per episode of care); a focus on the identification and brief treatment of common mental and behavioral health conditions of mild to moderate symptom severity and functional impairment; and referral to general or specialty mental health services for Veterans requiring longer-term, more intensive, or higher-acuity care. Each practice management category included sub-categories with specific items considered consistent with the MBHI Demonstration Projects aims. For example, the category for screening, referral to care, and follow-up included the sub-category of same-day open access. To align with expectations, MBHI sites must encourage and facilitate warm handoffs. Teams were encouraged to use this resource during pre-implementation and over time to review implementation progress and compliance with expectations.

Initial implementation support was provided by VHA Office of Mental Health Integrated Services and Center for Integrated Healthcare (CIH) Education & Implementation team for MBHI, which are referred to jointly as the MBHI Actions Team throughout this article. Support was readily available through email communications, one-on-one meetings (as requested), and through the development and dissemination of multiple resources designed to aid integrated care activities (e.g., implementation parameters, handouts, documentation templates, and flyers). Such support also included community of practice calls that alternated between technical assistance (e.g., clinic set-up requirements, funding) and education and training for MBHI teams (e.g., how to conduct and receive warm handoffs, communication and collaboration).

Program evaluation for the Demonstration Projects included initial site visits. These were completed for a random sample of participating facilities that had staff recruited or on board. Visits gathered information regarding: 1) early implementation experiences; (2) perceived benefits of MBHI services; (3) facilitators of program implementation; (4) areas for growth; and (5) barriers to implementation. Findings may inform program refinements, support scalability, and contribute to the review of best practices for broader implementation.

## Materials and methods

Analyses were conducted as part of ongoing operations and quality improvement work by the Serious Mental Illness Treatment Resource & Evaluation Center (SMITREC) in the Office of Mental Health. The present work constitutes an operations project rather than a research study and it did not require institutional review board approval (7).

### Site selection

A total of 53 VHA administrative parent facilities received funding to implement integrated mental and behavioral health care (including suicide prevention) in Pain and/or Oncology Clinics (45 for Pain Clinics, 29 for Oncology Clinics). Sites were informed of funding decisions in June of 2022, and hiring efforts began shortly thereafter. Of these, as of 3/24/2023 when selection of sites for visits occurred, there were 26 MBHI-Pain sites and 23 MBHI-Oncology sites which had either hired or were in the process of on-boarding one or more MBHI providers. Because some facilities received funding to hire MBHI providers in both Pain Management Teams and Oncology Clinics, these totals include overlap; there were 32 unique facilities with one or more MBHI providers hired or on-boarding. These program sites were categorized as follows: facilities with funded Pain Clinics only (*n* = 9); facilities with funded Oncology Clinics only (*n* = 6); and sites with both funded Pain Clinics and funded Oncology Clinics (*n* = 17).

From each of these three categories, three sites were randomly selected, for a total of nine sites being selected for program visits. The only criteria used in the random site selection process were (1) the type of clinic(s) for which the facility received Demonstration Projects funding (Pain Management Team only, Oncology Clinic only, or both) and (2) whether the facility had one or more MBHI providers hired or in the process of on-boarding at the time of site selection (3/24/2023). Given the relative recency of Demonstration Projects funding at the time of site selection and the limited number of facilities meeting these initial criteria, it was not feasible to further stratify random selection on the basis of facility patient population size, proportion of rural Veterans served, or other facility characteristics. However, the nine facilities visited were geographically dispersed, across eight regional areas referred to as Veterans Integrated Service Networks (VISNs). One served smaller Veteran populations (<25,000), and two served a greater than average proportion of rural Veterans (i.e., greater than approximately one-third of the patient population). Site visits were completed in October and November 2023, representing an early period of implementation defined as approximately 12 to 18 months after sites were notified of funding decisions in June 2022, and generally 6 to 12 months after MBHI providers were hired or on-boarded.

### Procedure

Site visits were completed in person over one to two days each. Site visit teams each included members of the MBHI Evaluation Team of SMITREC, and at one site included representation from a member of the Center for Integrated Healthcare Education & Implementation team for MBHI (due to geographic proximity and availability). Site visitor roles included program evaluators (e.g., health scientists, epidemiologists) and integrated care specialists. Each site was visited by one to three site visitors, and all nine site visits included at least one member of the SMITREC MBHI Evaluation Team.

E-mail correspondence regarding planned site visits included a mental health liaison for the VISN, facility-level MBHI Demonstration Projects implementation point-of-contacts (POCs), and other facility or VISN contacts depending on local protocols for coordinating site visits. Visit dates were selected based on the availability and preferences of specific sites, to avoid any disruptions to patient care. The duration of a typical visit was approximately eight hours in one day. For visits to sites that received funding to hire MBHI providers in both Pain and Oncology Clinics and/or sites funded for positions posted at multiple campuses, visits extended into two days to ensure time to visit both specialty clinics and/or locations. The pre-arranged agendas and schedules were coordinated with the POC(s) at each program, responsible for arranging meeting times and locations for visits (Supplementary Appendix A).

Each visit included: 1) an overview of the program given by the local program lead, other involved staff, and facility and/or VISN leadership; 2) meetings with Oncology Clinic and/or Pain Clinic staff to learn about MBHI services, care delivery, clinic processes, implementation, and plans; 3) tour of the physical location(s) of medical services and MBHI services delivery; 4) individual meetings with the Chiefs of Mental Health, Pain, and/or Oncology; 5) meetings with Pain and/or Oncology medical providers; meetings with MBHI provider(s); and 6) meetings with existing integrated mental health provider(s). Additional meetings at some sites included meetings with: Palliative Care leadership and/or providers; Physical Medicine and Rehabilitation (PM&R) leadership and/or providers; Chiefs of Social Work, Psychiatry, Behavioral Health, or Nursing; and/or national Pain consultants. Sites were advised that meetings with Veteran clinic patients were welcome but ultimately optional and at the facility's discretion. One visit included a meeting with a Veteran who had received program services, heard of the site visit, and expressed interest in meeting with the visitors.

Site visitors followed a general framework of questions to assure collection of similar types of information across sites, while seeking additional information pertinent to site-specific issues (Supplementary Appendix B). Content included: fidelity to core practice management components and basic model requirements (i.e., brief treatment model, same-day access, colocation) as well as site-specific modifications and procedures; types of patients and conditions addressed; program operation; perceived value and impact of MBHI services; degree of integration of MBHI providers into specialty medical setting team; program strengths and factors for successful and sustained implementation; program challenges and growth areas; future goals for integrated care and Suicide Prevention efforts; and perspectives about evaluation efforts and key aspects of program to track.

### Data analysis

Visitors’ notes were consolidated to generate individual site visit reports. These were then content coded, organized by evaluation question, and thematically analyzed. The evaluation team reviewed until consensus was reached for the summary report of key findings. These were categorized as: (1) perceived benefits, (2) strengths and facilitators for early implementation success, and (3) challenges, barriers, and areas of potential concern. Results present the percentages of the nine sites which reported information relevant to each theme and/or content code.

## Results

### Implementation of core practice management components of MBHI

MBHI services were available at all nine sites, and all were in the early period of implementation. Services were provided by one or more MBHI providers, and a majority of MBHI hires were a single individual contributing 1.0 FTE (see Table 1 for summary of core program components by site). Components of care outlined in Demonstration Project guidance (Supplementary Appendix C) were variably interpreted and applied across these nine sites, including adherence to a brief treatment model (which was either not present or with some difficulty noted at four of the six visited MBHI-Oncology sites and at three of the six MBHI-Pain sites). MBHI providers had dedicated availability for same-day access at all the MBHI-Oncology sites and at five of the six MBHI-Pain sites. Sites varied in the extent to which they had identified physical space for MBHI providers within specialty medical clinics and in integrating MBHI providers into their clinic teams. MBHI providers were physically colocated within the specialty medical clinic at least part of the time at 5 of the 6 MBHI-Oncology sites and at four of the six MBHI-Pain sites.

**Table 1:** Availability and adherence to core components of Mental and Behavioral Health Integration (MBHI) into pain management teams and oncology clinics Demonstration Projects.

Site	MBHI Provider(s) Hired and Onboarded	Number/Type of MBHI Provider(s)	MBHI Services Available within Clinic	Physically Colocated in Clinic	Same-Day Access Available	Brief Treatment Model Adherence
**Sites With MBHI-Pain and MBHI-Oncology**						
**#1 Pain**	Yes	Psychologist (1)	Yes	Yes (Part-time)	Yes	Yes
**#1 Oncology**	Yes	Psychologist (1)	Yes	Yes (Part-time)	Yes	Some Difficulty Noted
**#2 Pain**	Yes	Social Worker (1)	Yes	No	Yes	Some Difficulty Noted
**#2 Oncology**	Yes	Social Worker (1)	Yes	Yes (Part-time)	Yes	Some Difficulty Noted
**#3 Pain**	Yes	Psychologist (1) Social Worker (1) Psychiatrist (0.5)	Yes	Yes	Yes	Yes
**#3 Oncology**	Yes	Psychologist (2)	Yes	Yes	Yes	Yes
**Sites with MBHI-Pain Only**						
**#4 Pain**	Yes	Psychologist (2)	Yes	No	No	No
**#5 Pain**	Yes	Psychologist (0.5)	Yes	Yes (Part-time)	Yes	Some Difficulty Noted
**#6 Pain**	Yes	Psychologist (3) Psychiatrist (1)	Yes	Yes (Part-time)	Yes	Yes
**Sites with MBHI-Oncology Only**						
**#7 Oncology**	Yes	Psychologist (1)	Yes	Yes	Yes	Yes
**#8 Oncology**	Yes	Psychologist (1)	Yes	No	Yes	Some Difficulty Noted
**#9 Oncology**	Yes	Psychologist (1)	Yes	Yes	Yes	Some Difficulty Noted

Specific program parameters include same-day access availability and adoption of a brief treatment model. This includes initial assessments of 30 minutes or less focused on the primary reason for referral and its functional impact, as well as brief, time-limited follow-up interventions (typically ≤30 minutes and 1–6 sessions per episode of care). MBHI care is focused on the identification and brief treatment of common mental and behavioral health conditions of mild to moderate symptom severity and functional impairment, with referral to general or specialty mental health services for Veterans requiring longer-term, more intensive, or higher-acuity care. Several sites noted difficulty consistently adhering to a 30-minute session length standard for different reasons. Within Pain Clinics, mental health comorbidities were common and myriad, resulting in complex patient presentations. Within Oncology Clinics, MBHI providers discussed challenges balancing brevity of sessions with providing validation and compassion for Veterans with serious or terminal cancer diagnoses.

Visitors noted that teams and leadership at larger facilities demonstrated notable commitment to advancing integrated mental health care and understood the characteristics and elements that contribute to a successful model of integrated care. Staff at one site reported some initial uncertainty regarding key elements of the Demonstration Projects, including the intended focus on brief and time-limited care, same-day access, physical colocation, and collaboration with specialty medical team providers. Specific services offered by MBHI providers and patient populations served also varied to address the unique needs of the patient population of the facilities and/or clinics. Within Pain Management Teams, MBHI providers commonly delivered brief, time-limited psychotherapy for chronic pain, depression, anxiety, post-traumatic stress disorder (PTSD), and sleep disturbances. Therapeutic interventions included brief courses of cognitive behavior therapy for chronic pain (CBT-CP), CBT for insomnia, acceptance and commitment therapy, motivational interviewing, stress management, behavioral activation, hypnosis, as well as spinal cord evaluations. Within Oncology Clinics, MBHI providers addressed coping and adjustment to cancer diagnosis, distress associated with treatment and side effects, survivorship, grief, and comorbid mental health conditions, including through psychotherapy groups. Same-day access encounters most often addressed acute stress, changes in emotional baseline, or positive suicide screens. At some sites, across both settings education and support groups were present as well as Whole Health-centric behavioral offerings related to mood issues, tobacco use, weight management, and chronic pain. At the one site with an MBHI psychiatrist, services also included psychotropic medication management, medications for opioid use disorder, and evaluations for medical procedures.

All sites described one or more implementation benefits, barriers, and facilitators. The number and percentage of sites reporting content in each theme and contributing code are presented in Table 2. Whenever possible, illustrative direct quotes are included from MBHI providers and specialty medical clinic providers.

**Table 2:** Categories of implementation benefits, barriers, facilitators, and recommendations for the Mental and Behavioral Health Integration (MBHI) into pain management teams and oncology clinics Demonstration Projects.

Category	Site Count (%)
**Benefits**	**9** **(100)**
Benefits for Clinic Operations: *“It's Great Help for Them to Jump in with a Positive Screen”*	*9 (100)*
Managing Suicide Risk	9 (100)
Team Dynamics and Clinic Workflow	7 (77.8)
Mental Health Needs	7 (77.8)
Clinic Capacity	5 (55.6)
Clinic Accreditation	3 (33.3)
Benefits for Patients: *“It's Made a World of Difference*”	*9* *(100)*
Broad Benefits	9 (100)
Reducing Stigma of Mental Health Care	4 (44.4)
Treatment Engagement	3 (33.3)
Benefits for Specialty Medical Team Members and Providers: “*It Was a Sigh of Relief When the MBHI Provider Came on Board*”	*8* *(88.9)*
Support for Team	6 (66.7)
Education	6 (66.7)
Reduced Disruptions to Provider Schedules	4 (44.4)
Patient Relationships	2 (22.2)
Facility Operations: *“Patients Don't Want to Go to the Emergency Room*”	*4* *(44.4)*
VA Behavioral Mental Health Care	4 (44.4)
Community Care	2 (22.2)
**Strengths and Facilitators for Early Implementation Success**	**9** (**100)**
Buy-In and Support for Demonstration Projects: *“We’re Really Behind this Initiative*”	*9* *(100)*
Added Value and Appreciation	9 (100)
Provider Buy-In	8 (88.9)
Facility Leadership Buy-In	7 (77.8)
Specialty Medical Team Integration: “*We’re Delivering a Better Service for Veterans Together*”	*7* *(77.8)*
Colocation and Space	5 (55.6)
Interdisciplinary Approach to Care	5 (55.6)
Education for Team Members	4 (44.4)
Partnerships and Resources: *“Establish those Relationships with Other Teams*”	*7* *(77.8)*
Training Opportunities	4 (44.4)
Utilizing Available Resources	4 (44.4)
Partnership across Service Lines	3 (33.3)
Educating Key Partners	3 (33.3)
MBHI Provider Approach to Care and Background: *“We Are Everywhere, Sometimes All at Once*”	*6* *(66.7)*
Self-promotion of Services	5 (55.6)
Services Offered	4 (44.4)
Content Expertise	3 (33.3)
Prior Integrated Care Experience	3 (33.3)
Specialty Medical Clinic Approach to Care*: “Once I Saw How Easy It Was… I Continued to Send Referrals*”	*6* *(66.7)*
Clinic Workflow	5 (55.6)
Well-established Clinic	4 (44.4)
Measurement-based Care	3 (33.3)
**Challenges, Barriers, and Areas of Potential Concern**	**9** (**100)**
Physical Space Restrictions: “*You Need to Be Physically in the Space. It Shows You are Valued*”	*7* *(77.8)*
Clinic Competing Demands and Workflow: *“Can One Person Do It?*”	*8* *(88.9)*
Flexibility for MBHI Care Delivery	5 (55.6)
Unclear Division of Roles and Responsibilities	4 (44.4)
Meeting Demand for Long-Term Psychotherapy	4 (44.4)
Meeting Demand of Clinic Volume	3 (33.3)
Operational and Management Pressures: “*Don't Measure Value Just on Encounter Volume*”	*7* *(77.8)*
Workload and Productivity Demands	6 (66.7)
Provider Clinic Builds	2 (22.2)
Referrals and Patient Sources: *“Sometimes There is Lack of Words or Time*”	*7* *(77.8)*
Intended Patient Population	4 (44.4)
Combatting Mental Health Stigma	3 (33.3)
Insufficient Source of Referrals	3 (33.3)
Difficulty Integrating MBHI into Clinic: *“It's Not Just Crisis Management*”	*6* *(66.7)*
MBHI Provider Integration	5 (55.6)
Awareness of MBHI Services	4 (44.4)
Team Meetings	4 (44.4)
Hiring and Program Development: *“It's a New and Difference Service”*	*6* *(66.7)*
Recruitment Challenges and Staffing	4 (44.4)
Programs in Development	3 (33.3)

*n* *=* 9 sites. Categories are listed below bolded evaluation questions.

### Benefits of MBHI

#### Benefits for clinic operations: “It’s great help for them to jump in with a positive screen”

All sites discussed one or more improvements to specialty medical clinic operations. The most commonly reported benefit, which was reported by all sites, was an enhanced ability to identify and manage patient suicidal thoughts and behaviors, including effectively implementing and responding to positive mental health and suicide screens. Most sites noted improvements related to team dynamics and clinic workflow (78%). This included team cohesion, managing cases of patients with complex needs, and reducing unnecessary or inappropriate consults. Most sites also discussed increased identification of mental health needs, access to mental health services, and/or referrals to facilitate care (78%). Staff at more than half of visited sites discussed increased capacity to meet patient needs within the specialty medical clinic (56%), including seeing a higher volume of patients and reduced wait times for medical visits, which was facilitated by MBHI providers addressing mental and behavioral health needs that might otherwise disrupt or extend medical appointments. One site visited discussed the ability to extend the length of their time-limited pain program from three to four months. With the addition of the MBHI provider(s), a third of sites discussed the ability to retain, gain, or re-gain optional clinic accreditation with professional organizations by meeting quality care standards (33%).

“Having psychologists embedded in medical specialties is one of the best things we have done for suicide prevention.” – Medical Provider

#### Benefits for clinic patients: “It’s made a world of difference”

Providers and/or leadership at all sites stated that clinic patients were experiencing at least some benefit from the introduction of the MBHI provider(s) into the specialty medical clinic (100%). Nearly half discussed the value of MBHI for addressing stigma associated with seeking mental health care (44%). A third noted increased patient engagement in medical treatment (33%), such as involvement in treatment planning and reducing no-show appointments.

“With [the presence of the MBHI providers], the burden is now reduced, and Veterans can engage more with the chronic pain part of their treatment. If their mental health is suffering, you can't expect them to engage in classes.” – Medical Provider

#### Benefits for specialty medical team members and providers: “It was a sigh of relief when the MBHI provider came on board”

Most sites (89%) reported one or more benefits for specialty medical team members and providers. Sites reported that providers felt supported, experienced less burnout, and were more comfortable addressing mental health concerns with patients (e.g., distress, suicide risk; 67%). They also felt more educated and aware of mental health needs and resources available to them, including suicide screening competency (67%). Nearly half of sites described reduced disruptions to provider schedules from patient emergent mental health needs (e.g., acute distress, active suicidal ideation identified during a medical appointment) as these were now addressed by the availability of MBHI provider same-day access (44%). This included completing comprehensive suicide risk evaluations and escorting patients to receive urgent mental health care. MBHI provider responses to these needs typically included immediate clinical assessment, brief intervention, and coordination with specialty mental health when indicated, distinguishing them from social workers assigned to medical specialty clinics who often focus on care coordination, resource navigation, and psychosocial support rather than direct clinical mental health assessment and intervention. Some sites also reported improved communication and relationships between providers and patients (22%).

“Part of the job can be to acknowledge the distress of the medical team.” – MBHI Provider

#### Benefits for facility operations: “Patients don’t want to go to the emergency room”

Nearly half of sites visited discussed one or more downstream impacts for their VA facility (44%), including reducing strain or backlog of referrals for behavioral or mental health care in other settings (44%). These included PCMHI, general or specialty mental health, the emergency department, and mental health walk-in clinic. Some also endorsed a reduction in Community Care referrals and/or the ability to “repatriate” Veterans to receive VA care (22%).

“We used to have to rely on mental health urgent care for managing crises.” – Medical Provider

### Strengths and facilitators for early implementation success

#### Buy-in and support for demonstration projects: “We’re really behind this initiative”

Support for the Demonstration Projects and buy-in from at least one source (e.g., medical clinic providers and staff, leadership) was evident at all sites visited (100%). All sites expressed appreciation and recognition of the added value from having the MBHI provider(s) as members of the specialty medical team (100%). Support for the MBHI Demonstration Projects and buy-in about the need for the services offered was recognized among specialty medical clinic staff and/or service line leadership at nearly every site visited (89%), and among facility leadership at most sites visited (78%).

“Someone should be available to talk with any new patient, because the doctors are really busy and the Veterans can sometimes only process ‘cancer, cancer, cancer.’ Being able to say, ‘We have someone available to talk. This is a lot to handle.’ is helpful as a service.” – Medical Provider

#### Specialty medical team integration: “We’re delivering a better service for Veterans together”

Nearly all sites discussed one or more facilitators related to MBHI provider integration into the clinic teams (78%). More than half of sites (56%) noted physical colocation into the specialty medical clinic space as critical for patient care, warm hand-offs to MBHI providers, and for referrals. This included clinics that did not currently have dedicated office space for MBHI providers within the specialty medical clinic. At more than half of sites, implementation was reported to be supported by a well-integrated team and/or interdisciplinary approach to care (56%). This included routinely seeking input from the clinic team, attending huddles and interdisciplinary team meetings, and including MBHI providers in clinic marketing/introductory materials to normalize their services as a standard part of care available to all patients. Some sites discussed the benefits of the MBHI providers and implementation leads educating other providers in the clinic and/or facility to improve mental health literacy (44%). This included developing a standard operating procedure and preparing medical providers for how to respond to patient distress.

“[The MBHI provider] is just part of our program; [they are] just another link in our chain of hands (supporting Veterans).” – Medical Provider

#### Partnerships and resources: “Establish those relationships with other teams”

Most sites discussed one or more factors related to partnerships and utilizing available resources for successful MBHI implementation (78%). Nearly half of sites reported involving mental/behavioral health trainees (e.g., predoctoral psychology interns, postdoctoral fellows) to support care within the specialty medical clinic (44%). The availability of certain resources was also perceived to be helpful among providers and/or leadership (44%), including those from the MBHI Actions Team (e.g., regular technical assistance calls, the MBHI program-specific Sharepoint site), VA resource hubs, and consultation with MBHI providers participating in the Demonstration Projects at other sites. Some benefited from partnering with other programs and service lines in their facility to coordinate care and identify unmet needs of the patient population (e.g., Palliative Care, PM&R, and Whole Health; 33%). Certain sites discussed efforts to provide education to VA and community partners to enhance awareness of best practices, MBHI services offered, and improve quality of care received (33%).

“We are also able to assist clinicians in other areas too. So, it has a broad impact on care and in other areas.” – Medical Provider

#### MBHI provider approach to care and background: “We are everywhere, sometimes all at once”

More than half of sites noted one or more strengths related to MBHI provider approaches to clinic care and/or their background (67%). At 56% of sites, MBHI providers engaged in various activities to self-promote and market their services to providers and patients. Several developed brochures and/or flyers introducing themselves, MBHI services, and how to get in contact. Additionally, some noted reviewing the roster and charts of patients with upcoming clinic appointments to pre-identify those who might benefit from MBHI services. Some sites discussed the benefit of MBHI providers offering a variety of services for clinic patients (44%). These included Whole Health-centric behavioral offerings (e.g., tobacco use, weight management, chronic pain) and specific mental health treatment approaches [e.g., hypnosis, pre-surgical evaluations (e.g., for spinal cord stimulation), education and support groups].

At some sites (33%), implementation was aided by the MBHI provider(s) having subject matter expertise (i.e., related to pain or oncology) to better understand unique experiences and treatment needs of patients being served in the specialty medical setting. This background knowledge was from prior training and/or acquired through experience gained in their current MBHI role. Some sites also discussed the benefit of MBHI providers having prior experiences in integrated healthcare settings (33%), including PCMHI.

“[The MBHI provider] is a pain guru.” – Medical Provider

#### Specialty medical clinic approach to care: “Once I saw how easy it was… I continued to send referrals”

Implementation at several sites was facilitated by factors related to specialty medical clinic's approach to care (67%), including workflow and processes for identifying appropriate mental health referrals (56%). For example, some sites adopted co-disciplinary appointments for all new patients involving both medical and mental health providers, as well as the incorporation of psychosocial screening completed by nursing staff for all new patients. Certain sites benefited from the specialty medical clinic or program being well-established and in operation for many years (44%). This included facilities that had already adopted an integrated approach to care in other medical services, a facility that was a leader in educating community providers to best serve their Veteran population, and a facility that was a national training hub for pain treatment. Three sites incorporated patient reported outcome measures into routine clinic care (33%), including comprehensive self-report measures of medical and psychosocial needs to identify Veterans’ awareness and interest in certain clinic services available to them.

“We want [the medical providers] to send us [the Veterans] the moment the thought forms in their head that this patient would benefit from seeing us.” – MBHI Provider

### Challenges, barriers, and areas of potential concern

#### Physical space restrictions: “You need to be physically in the space. It shows you are valued”

Space constraints of the facility and/or specialty medical clinic often prevented consistent colocation of MBHI providers (78%). This included both an overall lack of physical space as well as a lack of space in the “right area,” which could be a source of tension between services sharing office space in the same suites. Accommodations typically included MBHI providers adopting a hybrid schedule and spending at least some of their tour working remotely. Additionally, or alternatively, MBHI providers occupied dedicated or rotating office space in other areas of the facility.

“Integrated models are 100% the way to go because out of sight is out of mind.” – Clinic Provider

#### Clinic competing demands and workflow: “Can one person do it?”

Nearly all sites endorsed one or more challenges related to clinic competing demands and clinic workflow (89%). This included difficulty consistently adhering to certain guidelines specified in MBHI providers’ scope of practice and the need to flexibly adapt care to meet patient needs (56%), especially adhering to a 30-minute session length standard. Within Pain Clinics, mental health comorbidities were common and myriad, resulting in complex patient presentations. Within Oncology Clinics, MBHI providers discussed challenges balancing brevity of sessions with providing validation and compassion for Veterans with serious or terminal cancer diagnoses. At some sites, there was a lack of clarity about MBHI provider(s) scope of practice and how their roles and services differ from and complement other mental/behavioral health providers within the specialty medical clinic (44%). This difficulty was most apparent within PMTs, where existing behavioral health providers assess and treat mental and behavioral health disorders and provide pain-management interventions, and where new providers were recently hired as part of other funding initiatives. In contrast to MBHI providers, who deliver brief, integrated services with a focus on same-day access and mental and behavioral health comorbidities, pain psychologists and other embedded behavioral health providers typically deliver longer-term and/or pain-specific behavioral interventions (e.g., CBT-CP) as part of comprehensive pain management.

At 44% of sites, there was perceived unmet demand for traditional long-term psychotherapy or specialty mental health care among patients in the clinic and/or facility. This often led to concerns that MBHI providers would face pressure to address these needs, despite such services being outside their defined scope of practice. Difficulty meeting the demand for certain non-mental health clinic services was also discussed at a third of sites (33%). This included wait times for medical services and/or limited access to certain clinic services (e.g., physical therapy, nutrition). These findings reflected broader concerns about clinic flow, capacity, and continuity at these sites which could have potential downstream implications for MBHI implementation and utilization (e.g., decreased availability of medical providers to engage in team-based care, over-reliance on consult-driven process, and increased burden on the team of providers). However, additional investigation is needed to document and understand the impact of these factors on MBHI.

“We have so many patients. Can one person do it?” – Medical Provider

#### Operational and management pressures: “Don’t measure value just on encounter volume”

Most sites discussed experiencing operational and management pressures (78%), with more than half reporting pressure from leadership to meet productivity demands (67%). Productivity pressure was most commonly reported to come from mental health service line leadership, to whom MBHI providers typically reported. This contributed to difficulty consistently adhering to implementation guidelines like limiting MBHI service delivery to only the intended patient population and participation in integrated activities that do not contribute directly to productivity metrics (e.g., joint/co-disciplinary appointments, non-workload team activities). Providers discussed providing care that supports the VA mission to serve patients flexibly “even if it's technically outside [implementation guidelines].” Leadership at some sites also discussed challenges related to MBHI provider clinic builds (22%), particularly a large burden for scheduling assistants to accommodate VA video connect (telehealth to non-VA location) care.

“It's important that you don't measure value just on encounter volume. You also need to look at accessibility, responsiveness, ease of mind for providers. There’s no substitute for that.” – Medical Provider

#### Referrals and patient sources: “Sometimes there is lack of words or time”

Most sites also discussed some implementation challenges regarding patient referrals to MBHI providers (78%). For example, there was some variability and uncertainty about the intended patient population for MBHI services (33%). At some sites, MBHI providers accepted referrals for patients seen outside the outpatient Pain Clinic and Medical Oncology Clinic. Referral sources for Veterans with chronic pain included PM&R, Neurosurgery, and Spinal Cord Injury Clinic. Referrals were often received from clinics where a cancer diagnosis was first made (i.e., prior to engaging with the Medical Oncology Clinic), as this was perceived to be a time of greater suicide risk.

Some sites (33%) reported that stigma associated with mental health needs and treatment reduced referral volume to the MBHI provider(s). At one site, the MBHI provider spent part of their workday providing services from an office of a mental health clinic in another building, potentially deterring Veteran access and care engagement. Some medical providers misunderstood MBHI care to only address patient acute distress, limiting referrals for comorbid general mental/behavioral health needs. Three sites discussed challenges in building up their patient caseloads (33%). This was perceived to be due, in part, to the scope of care being limited to brief, time-limited psychotherapy versus longer-term pain-specific behavioral health intervention that was in high demand. At one site, providers expressed ambivalence about MBHI as they felt pressed for time and were not convinced about the added value of time spent in their session facilitating a referral. Additionally, some sites noted an inconsistent approach by clinic staff to screening for distress or suicide that may limit reliable identification of Veterans who would benefit from MBHI care.

“Long-term, it may be helpful to have boots on the ground, for example in Radiation Oncology.” – MBHI Provider

#### Difficulty integrating MBHI into clinic: “It’s not just crisis management”

Challenges related to specialty medical team integration were discussed by most sites (67%), with more than half noting integration of the MBHI provider(s) and the interdisciplinary approach to care could be improved (56%). Site visits were conducted relatively early during implementation of the Demonstration Projects, and implementation was expected to improve as clinic teams gained familiarity and comfort with the MBHI provider(s). Clinic medical providers and other staff at certain sites had limited awareness of the full scope of services offered by the MBHI provider(s) (44%), most notably availability of same-day access. While MBHI providers at certain sites were invited to attend interdisciplinary team meetings, rounds, and/or team huddles, there were limited opportunities for MBHI provider(s) at 44% of sites to participate in these types of activities; at some sites team meetings did not regularly occur.

“We’re not just here on call if a person is in distress.” – MBHI Provider

#### Hiring and program development: “It’s a new and different service”

More than half of sites also discussed challenges related to hiring MBHI providers and medical program development (67%). This included recruiting, hiring, and retaining MBHI providers in the specialty medical clinic and in their facilities (44%). Contributing factors were identifying ideal candidates to deliver integrated care and understanding the unique needs of the patient population. Certain clinics were not only in early stages of implementation, but also undergoing changes related to specialty medical clinic processes (e.g., identifying patient sources, operational procedures, hiring necessary medical and support staff; 33%). This included relatively nascent specialty medical clinics and reorganizing approaches to clinic operations following COVID-19.

“It’s a new and different service.” – Medical Provider

## Discussion

### Effective implementation of MBHI services

Findings highlight the potential value of the MBHI Demonstration Projects and factors relevant for successful implementation. Effective services were offered by MBHI providers who were 1) fully integrated into specialty medical teams; 2) physically colocated; 3) perceived by medical providers as offering valuable services to patients; 4) receiving a steady source of referrals from team members; and 5) clearly committed to and passionate about serving Veterans. A wide array of implementation support strategies was offered by the MBHI Actions Team to enhance uptake, which will be more fully described in future publications. Implementation was further aided by MBHI provider self-promotion, a well-developed approach to clinic care, and partnership across service lines. Results may be leveraged to inform best practices and expand this initiative nationally.

### Perceived benefits of MBHI care

Across all sites, clinic team providers expressed appreciation and recognition of the added value of the MBHI provider(s). MBHI services were seen to enhance access to and initiation of mental health care, reduce stigma, and improve treatment engagement. This aligns with broader VA priorities to improve Veteran access to mental health services and increase likelihood of engagement (8). Findings support results from similar VA integrated care programs that deliver brief, colocated and collaborative care (i.e., PCMHI), suggesting interacting with integrated mental health providers reduces wait times and improves access to mental health care (9), increases engagement in specialty mental health treatment as needed (10) and subsequent mental health treatment engagement (11) and promotes treatment for common mental health conditions such as depression (12). Additionally, prior work suggests Veterans appreciate a multidisciplinary approach to PMT care as it is efficient and reduces the number of unnecessary appointments (13).

Evidence suggests successful implementation of integrated behavioral healthcare depends on a standardized workflow, group cohesion, and growth through education (14), and several sites noted improvement in operations, workflow, and team unity. Several sites specifically noted greater ability to identify and manage mental health needs, particularly suicide risk, among patients. This aligns with prior work demonstrating that PCMHI providers improve identification of primary care patients’ mental health needs (15), and PCMHI is a critical setting for assessing suicidal thoughts and behaviors (16). Furthermore, several sites also discussed the value of MBHI providers providing education and support to clinic team members, reducing their burnout and improving their relationships with patients. This aligns with prior literature suggesting pain provider well-being is enhanced by supportive relationships and working with a multidisciplinary team (17). Additionally, one Oncology Clinic participating in the Demonstration Projects reported that MBHI success and sustainment required investment in the team's well-being (18).

Some sites also discussed the downstream impact of MBHI on reducing strain and backlog in other mental health care settings, as well as a reduction in reliance on Community Care. This aligns with findings suggesting PCMHI engagement is associated with improved quality of psychotherapy and reduced reliance on general mental health services (19, 20). Additional support comes from findings that Veterans with PTSD who receive care from highly implemented PCMHI clinics are less likely to use high-cost inpatient and specialty mental health services (21). Improving fidelity and increasing access to the MBHI intended treatment model may therefore stimulate downstream benefits.

### Recommendations for addressing barriers and future directions

Several challenging aspects of program implementation may be helpful to consider when developing recommendations for best practices and future directions. First, *insufficient or inappropriate physical space* was noted by several sites as a barrier for integrated care, often preventing consistent colocation of MBHI providers. While recognizing the logistical challenges and broader demand for physical space in many VA facilities, prior evidence suggests physical colocation is a key component for building collaborative relationships between interdisciplinary team members (22).

In addition to exploring opportunities for more consistent colocation of MBHI providers, their *integration into clinic teams* may be supported by encouraging activities that develop and strengthen partnerships. Such activities include team meetings, review committees, and co-disciplinary appointments. Such activities, however, can be difficult to capture through currently available coding and tracking methods, an enduring challenge for integrated mental health programs. The emphasis on bookability noted at several sites may also impede participation in these valuable activities, which are not well captured by current workload reports. However, while some of these activities are not considered “bookable” time, they are valuable for integration into specialty medical teams and collaboratively address patient care needs.

The *buy-in, support, and investment of medical providers* was noted by most sites to be critical for early implementation success. Likelihood of clinic team members referring to the MBHI provider hinges on their belief that services will benefit patients and the ease with which the referral process can be incorporated into their standard approach to care, to reduce burden on specialty providers. It could therefore be beneficial to explore opportunities to support, educate, and gain perspectives from medical providers about integration into the team as early in the planning process as possible. This may require program leadership at the facility level to ensure medical staff have their support and approval for engaging in such education and associated skill-building. Providing testimonials from high performing sites to improve provider buy-in and encouraging training to increase comfort and proficiency with confronting behavioral/mental health change in patients may also be beneficial.

In addition to buy-in to *facilitate appropriate referrals*, it is also essential that providers have awareness and accurate understanding of MBHI care. Providers and leadership at more than half of sites visited expressed at least some confusion about the specific roles and responsibilities of MBHI providers and how they differ from those of traditional mental health providers. This is not unlike experiences shared during early PCMHI implementation. Strategies employed during PCMHI implementation to address role clarity – including robust educational offerings for medical teams, competency-based training requirements for PCMHI clinicians, standardized program parameters including compliance checklists, ongoing implementation support, and regular Community of Practice calls – may inform ongoing MBHI development, several of which are already underway through the MBHI Actions Team (2). It would be helpful to employ implementation interventions that utilize multiple methods and strategies for clarifying and demonstrating how MBHI care complements the existing landscape of mental health services offered within and/or available to specialty medical clinics, including co-occurring initiatives funded by partners.

Additionally, identification of appropriate referrals was aided by partnership with adjacent services (e.g., Palliative Care) and MBHI provider self-promotion. Unlike academic medical centers where faculty websites often introduce providers and services to patients and referring clinicians, VHA does not have an equivalent readily accessible platform. MBHI provider self-promotion efforts (e.g., developing brochures and flyers, attending and presenting at Pain and Oncology team meetings, huddles, and rounds) may help fill this gap and normalize MBHI as a standard part of specialty medical care. Other suggestions to facilitate referrals included: adopting routine roster and chart review for upcoming patient appointments; orientation groups for all new clinic patients to introduce MBHI services and their availability; standardizing VA distress screening procedures for cancer patients; and suicide screening during care transitions. Sharing effective implementation strategies observed at high-performing sites – including MBHI provider self-promotion activities – across sites through Community of Practice calls and other implementation supports may facilitate uptake by newer or lower-performing sites.

An additional challenge navigated was balancing patient-centered flexibility of MBHI care delivery with *demand for services outside the MBHI provider's intended scope of practice* (e.g., length and number of sessions). Based on the complex and acute mental health needs of some patients seen by specialty medical teams (particularly by PMTs), the suitably of the 30-minute session length standard adopted from PCMHI was discussed at nearly half of sites visited. This may warrant review to ensure there is greater understanding of the benefits of early intervention (e.g., before symptoms progress to a level requiring longer-term or more intensive mental health care or crisis intervention), ability to identify more subtle or less severe presentations that would benefit from consultation with an integrated mental health provider, stepped-care and shared decision making to effectively accommodate the range of needs of the patient population, as well as continued efforts to ensure patients have access to a full continuum of mental health care to address such varying needs.

The MBHI role was designed to offer population-based services that focus on same-day access, brief evidence-informed assessments and interventions, and brief episodes of care. Most providers require additional training to acquire the unique skills necessary to deliver these services with fidelity (23, 24). Within the context of PCMHI, embedding mental health professionals without additional training initially resulted in duplication of more traditional mental health services (25, 26). It is possible that concerns raised about the suitability of the 30-minute session may stem from providers’ lack of training or comfort in providing these types of services, as only a third of sites reported their MBHI provider(s) had prior experience in integrated healthcare settings (including PCMHI). This highlights the importance of ensuring MBHI providers are fully trained in core competencies associated with providing integrated care. Within VHA, competency training is currently required for PCMHI providers (25) but has not yet been made a requirement for MBHI team members though it is available to them.

Several sites also discussed the importance of *flexibility in the sources of referrals* to MBHI providers and suggested expanding the definition of the intended patient population. This was more common within Oncology as Veterans receive cancer diagnoses in a variety of clinics before connecting with Medical Oncology, representing a period of high risk for suicide. Additionally, several providers discussed the value of providing services as Veterans transition to palliative care or through cancer survivorship. MBHI providers within PMTs also questioned whether it was appropriate to accept referrals from pain-relevant settings like PM&R, “Interventional” Pain Clinics, and Pain Programs vs. Clinics. Given that these were Demonstration Projects with limited staffing funds and were designed to evaluate benefits of this specific approach to integrated care, the number of medical teams and clinics from which the MBHI accepted referrals were concomitantly limited. One challenge in promoting flexibility of referral sources is the focus on integrated care being team-based with the medical provider leading the shared treatment plan. Should an MBHI provider extend services to multiple ancillary clinics and therefore multiple providers, the necessary close communication and collaboration and shared treatment planning may become strained to the point where care is no longer truly integrated or collaborative. Exploration of recommended staffing levels (i.e., how many specialty medical teams a single MBHI provider may be able to support while maintaining fully integrated care) will inform guidance around when, how, and to what degree to expand sources of referrals. PCMHI staffing guidance may offer a reference point for considering MBHI staffing expectations.

Despite recruitment and on-boarding challenges noted by several sites, most discussed an *interest in additional staff to support MBHI operations*. In addition to increasing the number of MBHI providers delivering care in their specialty medical clinic, there was a desire for certain types of mental health professionals. These included nurse managers for care coordination, social workers, and (most notably) psychiatric prescribers. At the site visited where a psychiatrist was an MBHI provider, several medical providers discussed the profound benefit of their addition. They were particularly appreciative of this MBHI provider's ability to help navigate drug interactions for psychotropic medications, considerations for opioid prescriptions, and addiction potential. These results are supported by prior work in PCMHI, which found programs with greater overall PCMHI staffing had higher levels of psychotherapy receipt, and higher numbers of PCMHI social workers (who may fill medication care manager roles) were associated with greater receipt of adequate antidepressant medication (19).

Providers and leadership at sites expressed differing opinions about the *ideal training, background, and philosophy of care* for MBHI providers. Some emphasized the importance of adopting a generalist approach to meet a broad range of patient behavioral and mental health needs and increase the pool of applicants. Others, however, discussed the value of MBHI providers having an incoming or acquired background and subject matter expertise that facilitated evaluation and treatment of pain- or cancer-specific needs. For these specific Demonstration Projects, however, MBHI providers are expected to serve as integrated care generalists with specialty clinic-specific knowledge, skills, and abilities (KSAs). Accordingly, providers with background or training in integrated care focused on competent provision of brief services for common mental health and health behavior concerns are likely to be a strong fit for MBHI positions in any setting. To excel in this role, however, additional competence is needed related to medical concerns and conditions specific to the setting. It is likely that many MBHI providers may enter this position with certain strengths and areas for growth that require attention to support successful implementation of these Demonstration Projects. In light of this, the MBHI Actions Team has been piloting an integrated care competency-based training program to build and enhance the KSAs necessary for this type of work. Notably, it is not only the MBHI providers that require specific skills to successfully deliver integrated, team-based care; all members of the team must have such competencies. If not previously experienced with or trained in delivering integrated, team-based care, medical providers and other team members must also build necessary KSAs. This need is also being explored.

### Limitations and future directions

The present study had several strengths, most notably that site visits were: thorough and content derived was robust, contributing to knowledge about various aspects of care; occurred early on as sites were first implementing MBHI services; involved both group and one-on-one meetings with MBHI providers, medical clinic providers and staff, and program and facility leadership; and yielded meeting notes that were closely reviewed and thematically analyzed. Nonetheless, present findings should be interpreted within the context of several limitations. Site visits were conducted to a sample of facilities participating in the Demonstration Projects and were conducted during preliminary phases of implementation. While it is not feasible to travel to each funded clinic spanning 31 U.S. states and territories, sites visited varied on several characteristics (i.e., geographic location, facility size, number and type of MBHI providers); this may nonetheless limit generalizability of findings. We also note that although sites were geographically dispersed across eight VISNs and varied in facility size and MBHI provider composition, facility patient population size and rurality of Veterans served were not stratification criteria in the random site selection process due to the limited number of facilities meeting initial selection criteria at the time of site selection. This may limit generalizability of findings to facilities serving predominantly rural Veteran populations or to those at the extremes of facility size. Finally, sites were visited during early implementation of the MBHI Demonstration Projects, and experiences and procedures will likely change over time. Site visits were one component of a larger program evaluation and provide critical information to support formative program development.

Findings from these 2023 site visits were shared with leadership of participating sites, and a summary report was shared with implementation partners and policy leadership. Additionally, information gathered as part of the site visits will continue to inform the future of this initiative through an ongoing quality improvement process. Both identified facilitators and challenges will be considered and used to improve the initiative, allowing locations to capitalize on existing facilitators and work to minimize and mitigate identified barriers. These initial findings will continue to inform the type and content of training and implementation support offered. For example, additional strategies to enhance MBHI role clarity and gather medical provider buy-in have already been designed and offered. Further, an initial MBHI competency training program has been developed and piloted, with plans to continue to expand competency training over future years. Results will be further incorporated with other evaluation efforts to inform program improvement and potential expansion, with the goal of ensuring the best possible integrated mental and behavioral health care services are provided to Veterans within outpatient specialty medicine settings throughout the healthcare system.

## Data Availability

Data used for the current project are derived from VA sources and are not publicly available. Requests to access the datasets should be directed to John.McCarthy2@va.gov.
