# Supplementary material for: Integrating mental and behavioral health providers into VHA pain management teams and oncology clinics: assessment of early implementation from site visits
Source: Front Health Serv. 2026 Sep 1;6:1863653. doi: 10.3389/frhs.2026.1863653 (PMC13574793; doi:10.3389/frhs.2026.1863653)
Supplement: Supplementary file 1 [file Datasheet1.pdf]

Appendix A. Site Visit Agenda – One Day Visit

Time	Meeting Description	Attendees
8:30 – 9:00 AM<br>Location:	Arrival and Orientation<br>Purpose: Introduce the MBHI Demonstration Projects site visit team and orient to the day and location.<br>Evaluation team can provide an overview of the site visit objectives, solicit input, and review the visit agenda.	Visit Coordinator / MBHI Implementation Point-of-Contact (POC)<br>VAMC Director or Other Facility Leadership (if preferred)
9:00 – 10:00 AM<br>Location:	Group Meeting with MBHI Team<br>Purpose: Learn about MBHI services, program functions, processes, implementation, and plans.	MBHI Implementation POC(s)<br>MBHI Provider(s)<br>Pain or Oncology Providers
10:00 – 11:00 AM<br>Location:	Tour & Individual Meetings: MBHI Provider(s)<br>Purpose: Learn about the physical and virtual location(s) of services and MBHI service delivery.	MBHI Provider(s)
11:00 – 11:30 AM<br>Location:	Individual Meeting: Chief of Mental Health<br>Purpose: Obtain in-depth information regarding MBHI services, from the Chief's perspective.	Chief of Mental Health
11:30 – 12:00 PM<br>Location:	Individual Meeting: Chief of Pain or Oncology<br>Purpose: Obtain in-depth information regarding MBHI services, from the Chief's perspective.	Chief of Pain or Oncology
12:00 – 12:45 PM	Lunch/Break	
12:45 – 2:30 PM<br>Location(s):	Individual, Group Meetings: Pain or Oncology Providers<br>Purpose: Obtain information from providers about the process, perceptions, and effectiveness of integrated care services.	Pain Providers and Staff
2:30 – 3:15 PM<br>Location:	MBHI Evaluation Team Meeting<br>Purpose: Site visit team to consult together and prepare summary for meeting with leadership.	MBHI Evaluation Team
3:15 – 4:00 PM<br>Location:	Meeting with Facility Leadership<br>Purpose: Summarize perspectives from visit and obtain information and perspectives from facility leadership regarding MBHI.	VAMC Director<br>Chief Medical Health Officer<br>Chief of Mental Health<br>Chief of Pain or Oncology<br>MBHI Implementation Lead/POC(s)
4:00 – 4:30 PM<br>Location:	Exit Meeting with MBHI Leadership<br>Purpose: Review, address any remaining questions, gather final perspectives, and summarize visit.	MBHI Leadership<br>MBHI Implementation Lead/POC(s)
