# Supplementary material for: Integrating mental and behavioral health providers into VHA pain management teams and oncology clinics: assessment of early implementation from site visits
Source: Front Health Serv. 2026 Sep 1;6:1863653. doi: 10.3389/frhs.2026.1863653 (PMC13574793; doi:10.3389/frhs.2026.1863653)
Supplement: Supplementary file 2 [file Datasheet2.pdf]

MENTAL/BEHAVIORAL HEALTH INTEGRATION (MBHI) INTO PAIN CLINICS AND ONCOLOGY CLINICS DEMONSTRATION PROJECTS

MBHI EVALUATION

SITE VISIT

draft version 10-16-23

Contents

Section 2: Site Visit Materials 2
Visit Structure 2
I. Arrival and Orientation Meeting 2
II. Group Meeting with MBHI Staff 3
III. Tour of settings of MBHI services 5
IV. Individual Meetings with MBHI Clinicians
Pain Clinic: Psychologists, Psychiatrists, and/or Social Workers 6
V. Individual Meetings with MBHI Clinicians
Oncology Clinic: Psychologists, Psychiatrists, and/or Social Workers 8
VI. Individual Meetings with MBHI Staff: Existing Mental Health Provider 10
VI. Individual Meetings with MBHI Leadership 11
VII. Individual Meetings with Leadership 12
VIII. Individual Meetings with Leadership 14
IX. Individual Meetings with Providers 16
X. Individual Meetings with Providers 17
XI. Site Visitor Review Meeting 18
XII. Summary Meeting(s) with Facility Leadership 19
XIII. Exit Meeting with MBHI Leadership 19

Section 2: Site Visit Materials

Visit Structure

I. Arrival and Orientation Meeting

Purpose: Introduce the MBHI Evaluation site visit team and orient the visitors to the day and location. Visitors can provide an overview of the site visit objectives, solicit input, and review the visit agenda.

Recommended attendees: Visit coordinator or MBHI Implementation Point-of-Contact (POC), VAMC Director or other facility Leadership (if preferred).

- Thank group for hosting the visit.
- Introductions of facility staff and site visit team members
- Describe the role of MBHI Evaluation
    - Assess early implementation, including barriers and successes
    - Inform implementation efforts, including future broader adoption of MBHI providers in other medical settings
    - Evaluate short-term and long-term goals, priorities, and needs
- Explain to group our reporting process
    - Provide facility leadership with feedback at end of visit
    - Observations will contribute to detailed summary impressions for national MBHI partners describing early program implementation experiences, potential best practices

a. Questions or Concerns:

b. Define MBHI (*Ask for their definition/conceptualization of initiative at their site*):

c. Are MBHI services currently available at this facility (very brief in this section):

1. Co-location of MBHI provider(s): Yes / No
2. Integrated/collaborative care: Yes / No
3. Providing care to pain clinic (420) or medical oncology (316) patients: Yes / No

DRAFT

II. Group Meeting with MBHI Staff

Program Organization

1. **What were your clinic's goals in applying for this Demonstration Project?**

2. **Is the site currently offering and/or providing MBHI services to its patient population?**
    a. *If yes, when did this provider begin?* Click here to enter a date.
    b. *If yes, are your clinic's goals currently being met?*

    c. *If no, why are MBHI services not currently available?*

3. **How were mental health needs of clinic patients previously addressed? Was mental or behavioral health care offered within the clinic prior to MBHI?**
    a. *If yes, by whom? Were they co-located?*

    b. *To address what need?*

4. **Does this facility provide MBHI services for other VAMCs, campuses, or CBOCs?**
    a. *If yes, by what means and by what schedule?*

5. **How is specialty medicine care (i.e., oncology or pain) organized in this facility?**
    a. *Coordination with other services* (e.g., SUD and tertiary pain clinics; palliative care)

    b. *Means of delivering medical and mental health care* (e.g., telehealth, F2F, hybrid schedule)

        i. *Is telehealth offered to address distance/rurality of patient population?*

    c. *Number and type(s) of MBHI provider*

    d. *Number and type(s) of non-mental health providers in clinic*

    e. Number and type(s) of support staff

Referral Process and Clinic Workflow

6. **How are referrals for MBHI providers identified in the clinic? What works well?**

7. **Is the MBHI provider on-site, completely remote, or hybrid?**

8. **How is care organized and expected to work?**

    a. *Who is the intended Veteran population* (e.g., diagnoses, source of referral, acuity)?

b. *What is the average wait time for a Veteran to be seen by a MBHI provider for a scheduled initial or follow-up appointment?*

c. *How is same-day access being organized/managed?*

d. *How is initial and follow-up treatment being organized/managed* (e.g., length of sessions, number of sessions)?

9. **What are expectations and workflow for mental health screenings (including suicide) and response to positive screens?**

10. **What are the specific modes of communication between providers** (e.g., Teams/email, curbside consultation, non-encounter work, IDT meetings, huddles, rounds, shared appointments)?

11. **How are MBHI providers integrated into treatment planning for Veterans**?

12. **How are things generally going?**
    a. *What barriers remain to full program implementation?*

Program Impact

13. **How have MBHI services affected pain/oncology patients (e.g., needs, expectations)?**

14. **How have MBHI services affected pain/oncology providers (e.g., needs, expectations)?**

15. **How does specialty mental health interface with MBHI? What is the mode of communication?**

16. **What performance metrics are being used locally to track implementation/impact?**

17. **What are the strengths and advantages of the MBHI program:**

18. **What are the barriers and challenges, if any, facing the program:**
    ⬭ Staffing ⬭ Lack of Training ⬭ Cultural Shift in Care Needed ⬭ Other

19. **Early successes and lessons learned from program:**

20. **Suggestions, next steps, or "wish list" for MBHI:**
    a. *Metrics to measure success of implementation and outcomes?*
    b. *Metrics to include in Dashboard?*

21. **What resources are needed for you to achieve your clinic's goals?**

22. **Comments for Leadership:**

23. **Pre-Work Concerns:**

DRAFT

III. Tour of settings of MBHI services

a. **Location of Oncology/Pain Clinics:**

b. **Location of MBHI provider:**

c. **Is access compromised due to the location of the oncology/pain clinic and MBHI provider?**

d. **Is the MBHI provider's office location stable or rotating?**

e. **Challenges of the MBHI provider's location:**

f. **What is the process with a warm hand-off?**

    a. *What about when the MBHI provider is busy?*

g. **What is the average wait time for a Veteran to be seen by a MBHI provider for a same day appointment?**

h. **Do Veterans mind waiting for the MBHI provider, where do they wait, and do some Veterans leave?**

DRAFT

IV. Individual Meetings with MBHI Clinicians

Pain Clinic: Psychologists, Psychiatrists, and/or Social Workers

a. **What is your role(s) in the pain service?**

b. **Hiring experience (e.g., new to VA; new to integrated care in medical setting)?**
   a. *Did you have experience providing integrated care in other settings (e.g., PCMHI)?*

c. **What is your schedule?**
   a. *What percentage of your time is spent in the pain clinic? Where else?*

   b. *What percentage of your tour is telework (if any)?*

d. **How satisfied are you with your position/job?**

e. **Processes & Procedures of Co-located, Integrated Care**
   1. *Referrals*
      a. Patient population seen (including % of newly diagnosed)

      b. Sources and system for referrals

   2. *Same-day access*
      a. How is your open access organized (e.g., same day availability hours)

      b. How often is there a warm hand-off between you and the medical provider?

      c. Is the Veteran usually present for the warm hand-off?

   3. *MH/BH Diagnosis and Intervention Delivery*
      a. Length and number of sessions

      b. Conditions treated, common presenting problems

      c. Coordination/consultation with SMH/PC, continuity of care

      d. Types of services offered (e.g., EBTs, couples/family, groups)

   4. *Screening*
      a. MH screens (including SUD) completed and by whom

      b. Suicide screening and SOP re: responding to positive screens

   5. *Case management, outreach, and follow-up (i.e., referral to specialty MH, continuity of care)*

DRAFT

6. *What has been your experience with co-location? Any issues with space?*

7. *Telehealth vs. face-to face services offered*

8. *How do you interact with the other providers? Is there true integration into clinic team?*

    a. Existing MH/BH providers?

    b. Joint appointments?

    c. How is information shared between providers?

    d. Participation in IDT, rounds, huddles, other meetings?

    e. How does telework/hybrid schedules impact this?

9. *What kinds of data are being collected (i.e., PROMs and MBC)*

10. *Other responsibilities (supervising trainees, didactics and education, committees, etc.)*

f. **Is MBHI valued here? Do you feel supported?**

g. **What are the strengths and advantages of the of MBHI program, from your perspective?**

h. **Any concerns? What are the barriers and challenges, if any, facing the MBHI program?**
   ⬭ Staffing ⬭ Lack of Training ⬭ Cultural Shift in Care Needed ⬭ Other

i. **What should we tell leadership at the end of the day about the program?**

j. **What should the future of MH integration in pain be? Future of MBHI?**

k. **Specific goals for the evaluation, recommendations/requests for metrics to measure success of MBHI implementation and outcomes (including Dashboard):**

l. **Comments for Leadership:**

m. **Pre-Work Concerns:**

DRAFT

V. Individual Meetings with MBHI Clinicians

Oncology Clinic: Psychologists, Psychiatrists, and/or Social Workers

a. **What is your role(s) in the oncology service?**

b. **Hiring experience (e.g., new to VA; new to integrated care in medical setting)?**
   a. *Did you have experience providing integrated care in other settings (e.g., PCMHI)?*

c. **What is your schedule?**
   a. *What percentage of your time is spent in the oncology clinic? Where else?*

   b. *What percentage of your tour is telework (if any)?*

d. **How satisfied are you with your position/job?**

e. **Processes & Procedures of Co-located, Integrated Care**
   1. *Referrals*
      a. Patient population seen (including % of newly diagnosed)

      b. Sources and system for referrals (e.g., Medical vs. radiation, surgical oncology)

   2. *Same-day access*
      a. How is your open access organized (e.g., same day availability hours)

      b. How often is there a warm hand-off between you and the medical provider?

      c. Is the Veteran usually present for the warm hand-off?

   3. *MH/BH Diagnosis and Intervention Delivery*
      a. Length and number of sessions (and location, e.g., chemotherapy suite)

      b. Conditions treated, common presenting problems

      c. Coordination with other services (e.g., SMH/palliative), continuity of care)

      d. Types of services offered (e.g., EBTs, Caregiver, groups; managing medical FX)

      e. Comfort level with delivery of psychosocial treatment

   4. *Screening*
      a. MH screens completed and by whom

      b. Distress screenings and by whom

      c. Suicide screening and SOP re: responding to positive screens

DRAFT

5. *Case management, outreach, and follow-up (i.e., referral to specialty MH, continuity of care)*
6. *What has been your experience with co-location? Any issues with space?*
7. *Telehealth vs. face-to face services offered*
8. *How do you interact with the other providers? Is there true integration into clinic team?*
    a. Joint appointments?
    b. How is information shared between providers?
    c. Participation in IDT, rounds, huddles, other meetings?
    d. How does telework/hybrid schedules impact this?
9. *What kinds of data are being collected (i.e., PROMs and MBC)*
10. *Other responsibilities (supervising trainees, didactics and education, committees, etc.)*

f. **Is MBHI valued here? Do you feel supported?**

g. **What are the strengths and advantages of the of MBHI program, from your perspective?**

h. **Any concerns? What are the barriers and challenges, if any, facing the MBHI program?**
◯ Staffing ◯ Lack of Training ◯ Cultural Shift in Care Needed ◯ Other

i. **What should we tell leadership at the end of the day about the program?**

j. **What should the future of MH integration in oncology be? Future of MBHI?**

k. **Specific goals for the evaluation, recommendations/requests for metrics to measure success of MBHI implementation and outcomes (including Dashboard):**

l. **Comments for Leadership:**

m. **Pre-Work Concerns:**

DRAFT

VI. Individual Meetings with MBHI Staff: Existing Mental Health Provider

a. **What is your role(s) in the oncology/pain service?**

b. **How satisfied are you with your position/job?**

c. **Prior to MBHI, was there integrated care delivered in your clinic?**
   a. *Did you have experience providing integrated care in other settings (e.g., PCMHI)?*

d. **What type of services did you provider to patients before MBHI?**
   a. *How has this changed since the implementation of MBHI?*

e. **Describe working alongside the new MBHI provider (e.g., approach to patient care, responsibilities)**
   a. *Who performs mental health/suicide screens and comprehensive risk assessments?*
   b. *Who is responsible for same-day appointments? For scheduled initial and follow-up?*

f. **How do you interact with the other providers? Is there true integration into clinic team?**

g. **How has MBHI affected provider experiences?**
   a. *Workload and burnout, turnover*
   b. *Response to re-organization of care*

h. **How has MBHI affected care for clinic patients?**
   a. *Access to care*
   b. *Community care referrals*
   c. *Quality of care*
   d. *Population served*

i. **Is MBHI valued here? Do you feel supported?**

j. **What are the strengths and advantages of the of MBHI program, from your perspective?**

k. **Any concerns? What are the barriers and challenges, if any, facing the MBHI program?**
   ⬭ Staffing ⬭ Lack of Training ⬭ Cultural Shift in Care Needed ⬭ Other

l. **What should we tell leadership at the end of the day about the program?**

m. **What should the future of MH integration in pain/oncology be? Future of MBHI?**

n. **Specific goals for the evaluation, recommendations/requests for metrics to measure success of MBHI implementation and outcomes (including Dashboard):**

o. **Comments for Leadership:**

p. **Pre-Work Concerns:**

DRAFT

VI. Individual Meetings with MBHI Leadership

Chief of Mental Health

a. **What role does your leadership play in MBHI?**

b. **How do you communicate with other MBHI leadership partners?**

c. **Thoughts about promise/potential of MBHI, integrated health in medical settings**
   - *For suicide prevention, specifically*

d. **What do you see as the future direction for MBHI at this facility?**

e. **Has MBHI cut demand on SMH?**

f. **Has the patient population seen in SMH significantly changed?**

g. **Is MBHI valued in this facility (e.g., is there buy-in)? Do you feel supported (i.e., from local leadership in facility, national sources of support)?**

d. **Strengths of MBHI program:**

e. **Challenges of program (e.g., barriers to implementation):**
   ⬭ Staffing ⬭ Lack of Training ⬭ Cultural Shift in Care Needed ⬭ Other

f. **Lessons learned from program (successes/failures you've observed since implementation)**

g. **What should the future of MH integration in pain/oncology be? Future of MBHI?**

h. **Resources needed now and for the future:**

i. **Specific goals for the evaluation, recommendations/requests for metrics to measure success of MBHI implementation and outcomes (including Dashboard):**

j. **Comments for Leadership:**

k. **Pre-Work Concerns:**

DRAFT

VII. Individual Meetings with Leadership

Chief of Pain

a. **What role does your leadership play in MBHI?**

b. **How did your site become aware of the MBHI RFA/funding?**
   - *Why did your site decide to apply?*
   - *What are your goals for this provider/model of care? For suicide prevention?*
   - *What was the application process like?*
   - *What was the hiring/onboarding process like?*

c. **How do you communicate with other MBHI leadership partners?**

d. **What was pain care like before MBHI (e.g., when was clinic opened, growth, issues faced, reliance on external resources)?**
   - *How was the design of care developed?*
   - *What types of MH/BH care/providers existed in the pain clinic prior to MBHI?*

e. **How does the pain service feel about new dedicated MBHI providers taking up space?**

f. **Do you feel that MBHI providers decrease the workload for pain providers?**

g. **Is there a sufficient number of MBHI providers to meet pain MH needs?**

h. **Can integration be improved in the pain clinic?**

i. **Do MBHI providers have to promote themselves in the clinic?**

j. **What is the understanding and opinion of pain providers about MBHI services (including referrals, same-day access, warm hand-offs)?**

k. **Are MBHI providers fully integrated into the pain team? Do they attend pain staff meetings or other meetings (e.g., IDT, rounds, STORM, OSI, Opioid Safety Risk Reviews, Review of Community Care prescriptions)?**

l. **Is MBHI valued in this facility (e.g., is there buy-in)? Do you feel supported (i.e., from local leadership in facility, national sources of support)?**

m. **Strengths of MBHI program:**

n. **Challenges of program (e.g., barriers to implementation):**
   ⬭ Staffing ⬭ Lack of Training ⬭ Cultural Shift in Care Needed ⬭ Other

o. **Lessons learned from program (successes/failures you've observed since implementation)**

p. **What do you see as the future direction for MBHI at this facility?**

q. **Is integrated care the general direction the facility is going (e.g., integrated health in other settings)?**
   - *What about for suicide prevention, specifically?*

r. **Resources needed now and for the future:**

s. **Specific goals for the evaluation, recommendations/requests for metrics to measure success of MBHI implementation and outcomes (including Dashboard):**

t. **Comments for Leadership:**

u. **Pre-Work Concerns:**

DRAFT

VIII. Individual Meetings with Leadership

Chief of Oncology

a. **What role does your leadership play in MBHI?**

b. **How did your site become aware of the MBHI RFA/funding?**
   - *Why did your site decide to apply?*
   - *What are your goals for this provider/model of care? For suicide prevention?*
   - *What was the application process like?*
   - *What was the hiring/onboarding process like?*

c. **How do you communicate with other MBHI leadership partners?**

d. **What was oncology care like before MBHI (e.g., when was clinic opened, growth, issues faced, reliance on external resources)?**
   - *How was the design of care developed?*
   - *What types of MH/BH care/providers existed in the oncology clinic prior to MBHI?*

e. **How does the oncology service feel about dedicated MBHI providers taking up space?**

f. **Do you feel that MBHI providers decrease the workload for oncology providers?**

g. **Is there a sufficient number of MBHI providers to meet oncology MH needs?**

h. **Can integration be improved in the oncology clinic?**

i. **Do MBHI providers have to promote themselves in the clinic?**

j. **What is the understanding and opinion of oncology providers about MBHI services (including referrals, same-day access, warm hand-offs)?**

k. **Are MBHI providers fully integrated into the oncology team? Do they attend oncology staff meetings or other meetings (e.g., IDT, rounds)?**

DRAFT

l. **Is MBHI valued in this facility (e.g., is there buy-in)? Do you feel supported (i.e., from local leadership in facility, national sources of support)?**

m. **Strengths of MBHI program:**

n. **Challenges of program (e.g., barriers to implementation):**

⬭ Staffing ⬭ Lack of Training ⬭ Cultural Shift in Care Needed ⬭ Other

o. **Lessons learned from program (successes/failures you've observed since implementation)**

p. **What do you see as the future direction for MBHI at this facility?**

q. **Is integrated care the general direction the facility is going (e.g., integrated health in other settings)?**

- *What about for suicide prevention, specifically?*

r. **Resources needed now and for the future:**

s. **Specific goals for the evaluation, recommendations/requests for metrics to measure success of MBHI implementation and outcomes (including Dashboard):**

t. **Comments for Leadership:**

u. **Pre-Work Concerns:**

DRAFT

IX. Individual Meetings with Providers

Pain Medical Provider

a. **What is your role(s) in the pain service?**
b. **How satisfied are you with your position/job?**
c. **Before MBHI, how were your patients' MH needs addressed?**
d. **Prior to MBHI, was there integrated care delivered in your clinic?**
    a. *If yes, were providers co-located?*
    b. *Did you have experience providing integrated care in other settings (e.g., PCMHI)?*
e. **How has MBHI affected provider experiences?**
    a. *Workload and burnout, turnover*
    b. *Response to re-organization of care*
f. **How has MBHI affected care for clinic patients?**
    a. *Access to care*
    b. *Community care referrals*
    c. *Treatment adherence, appointment attendance*
    d. *Quality of care*
    e. *Population served*
    f. *OUD/MOUD screening and treatment*
g. **How do you communicate/consult with the MBHI provider to coordinate care for your Veterans (e.g., Teams/email, shared appointments, curbside consults, IDT meetings, rounds, huddles)?**
    a. *How does the "warm hand-off" work in your clinic?*
    b. *Do you trust recommendations from the MBHI provider?*
h. **Are the MBHI services valuable to you? Do you feel supported?**
i. **What are the strengths and advantages of the of MBHI program, from your perspective?**
j. **Any concerns? What are the barriers and challenges, if any, facing the MBHI program?**
    ⬭ Staffing ⬭ Lack of Training ⬭ Cultural Shift in Care Needed ⬭ Other
k. **What should we tell leadership at the end of the day about the program?**
l. **What should the future of MH integration in pain/oncology be? Future of MBHI?**
m. **Specific goals for the evaluation, recommendations/requests for metrics to measure success of MBHI implementation and outcomes (including Dashboard):**
n. **Comments for Leadership:**
o. **Pre-Work Concerns:**

DRAFT

X. Individual Meetings with Providers

Oncology Medical Provider

a. **What is your role(s) in the oncology service?**
b. **How satisfied are you with your position/job?**
c. **Before MBHI, how were your patients' MH needs addressed?**
d. **Prior to MBHI, was there integrated care delivered in your clinic?**
    - *a. If yes, were providers co-located?*
    - *b. Did you have experience providing integrated care in other settings (e.g., PCMHI)?*
e. **How has MBHI affected provider experiences?**
    - *a. Workload and burnout, turnover*
    - *b. Response to re-organization of care*
f. **How has MBHI affected care for clinic patients?**
    - *a. Access to care*
    - *b. Community care referrals*
    - *c. Treatment adherence, appointment attendance*
    - *d. Quality of care*
    - *e. Population served*
    - *f. Family/Caregiver support*
g. **How do you communicate/consult with the MBHI provider to coordinate care for your Veterans (e.g., Teams/email, shared appointments, curbside consults, IDT meetings, rounds, huddles)?**
    - *a. How does the "warm hand-off" work in your clinic?*
    - *b. Do you trust recommendations from the MBHI provider?*
h. **Are the MBHI services valuable to you? Do you feel supported?**
i. **What are the strengths and advantages of the of MBHI program, from your perspective?**
j. **Any concerns? What are the barriers and challenges, if any, facing the MBHI program?**
    ⬭ Staffing ⬭ Lack of Training ⬭ Cultural Shift in Care Needed ⬭ Other
k. **What should we tell leadership at the end of the day about the program?**
l. **What should the future of MH integration in pain/oncology be? Future of MBHI?**
m. **Specific goals for the evaluation, recommendations/requests for metrics to measure success of MBHI implementation and outcomes (including Dashboard):**
n. **Comments for Leadership:**
o. **Pre-Work Concerns:**

DRAFT

XI. Site Visitor Review Meeting

**Summary Site Visit Comments**<br>***(Site Visitors: please record overarching comments here to be included in your exit report for this area)***
**Strengths of Program and Services Offered:**
**Barriers, Limitations, Concerns, or Areas that Need Improvement:**
**Suggestions for Remedies:**
**Feedback to be Shared with MBHI Actions Team:**
**Feedback to be Shared with National Leadership/SPP:**
**Lessons Learned for Evaluation Efforts:**

DRAFT

XII. Summary Meeting(s) with Facility Leadership

a. Discuss strengths of MBHI care, services, and program at facility

b. Discuss barriers, limitations, and potential concerns of MBHI program at facility

c. Review suggestions and resources identified that may be needed for successful/improved implementation of MBHI

d. Discuss lessons learned that will be shared with national leadership (Jen's team and/or national leadership in SPP or elsewhere)

XIII. Exit Meeting with MBHI Leadership

DRAFT
