# Supplementary material for: Integrating mental and behavioral health providers into VHA pain management teams and oncology clinics: assessment of early implementation from site visits
Source: Front Health Serv. 2026 Sep 1;6:1863653. doi: 10.3389/frhs.2026.1863653 (PMC13574793; doi:10.3389/frhs.2026.1863653)
Supplement: Supplementary file 3 [file Datasheet3.pdf]

Appendix C.

Program Parameters for the Implementation of Mental/Behavioral Health Integration (MBHI) Demonstration Projects into Specialty Medicine Settings

**1. Population**	
1.1 Population of Focus for Care Provision	• Veterans are enrolled and *actively* engaged in care with the specialty medical team<br>• Veterans are not engaged in other mental health (MH) services
**2. Screening, Referral to Care, and Follow-up**	
2.1 Approach to Access	• Ensures daily same-day open access *and* sustained access for follow-up care through multiple same day access slots
2.2 Screening and Evaluation	• Systematic screening and tracking by all providers of key physical and behavioral health conditions<br>• Procedures for follow-up and coordination of positive screens are documented and shared with all applicable staff/providers<br>• The treatment team shares equal responsibility in Risk ID screening<br>• MBHI provider supports the medical team in completion of secondary risk screening and intervention, when indicated and appropriate
2.3 Same-Day Open Access	• Encourages and facilitates warm handoffs and engages in shared medical appointments or conjoint sessions<br>• Initial assessments are 30min or less<br>• Initial assessments are focused on the main reason(s) for referral with attention to how the primary concern is impacting functioning
2.4 Sustained Access (Follow-Up)	• Interventions are intended to be brief (≤30min) and time limited (1-6 sessions), per episode of care<br>• Co-facilitates shared medical appointments or conjoint sessions
2.5 Referral to Care	• Facilitates and supports a smooth transition across the system of care, when indicated and agreed upon
2.6 Consultation	• Provides and seeks consultation and education with the health care team to improve knowledge and skills in addition to promoting a team-based Whole Health approach to care<br>• Provides and seeks consultation and education with MH colleagues to improve understanding of roles and collaboration and coordination of care<br>• Facilitates consultation with or coordination of care with psychopharmacology services, when appropriate
**3. Treatment of Common Conditions**	
3.1 Use of Measurement Based Care	• Patient Reported Outcome Measures (PROMs) should place a premium on (a) brevity, (b) high reliability and validity, (c) relevance to the Veteran's primary concern, and (d) suitability to the specialty medical environment

	• Within service delivery, collection of PROMs should occur, at a minimum, at the initial visit, at least once during the course of treatment, and at the conclusion of treatment<br>• PROM results are integrated with additional data sources (e.g., Veteran report, clinician observations) and shared with the Veteran patient and treatment team<br>• PROM results and feedback are used to inform treatment planning
3.2 Mental Health-Specific Stepped Care Considerations	• Mild to moderate symptom severity and functional impairment<br>• Acute MH concern or stable chronic mental health concern
3.3 System/Local Resource Stepped Care Considerations	• Accept all referrals for initial brief assessment and consultation with understanding that Veterans will be referred to appropriate levels of care along the continuum of care
3.4 Use of Treatment Guidelines and Protocols	• MBHI interventions are population specific *and* brief (e.g., Brief CBT-Chronic Pain, Brief CBT-Insomnia, Problem Solving Therapy)<br>• MBHI interventions *may not be* population-specific *but* are brief (e.g., Brief CBT, tobacco cessation, Motivational Interviewing) *and supports continuity of care* in the setting where the Veteran has the *most treatment engagement*<br>• Utilizes available Clinical Practice Guidelines to inform treatment recommendations and promotes health behavior change in support of the integrated care team plan
3.5 Diagnosis/es of Focus for MHI Care Provision	• Encounters typically include medical and/or mental health diagnoses<br>• Encounters with a medical primary diagnosis utilize appropriate health and behavior procedure coding
**4. Health Behavior Knowledge and Skills**	
4.1 Health Behavior Knowledge and Skills	• General mental health *and* specialty specific knowledge and skills related to the medical clinic focus area (note, specialty specific training may occur post-selection/hire with development of population specific expertise over time)<br>• Utilizes an evidence-based approach to behavior change aligned with the biopsychosocial model of care<br>• Promotes and applies a Whole Health approach, ensuring team collaboration that is focused on what is most important to the Veteran
**5. Patient Health and Well-being**	
5.1 Promotion of Patient Health and Well-Being	• Provides education and support designed to empower, equip, and promote self-care and self-management skills based on the patient's personal preferences and priorities<br>• Educational tools are available via various modalities (e.g., handouts, web-based, mHealth-based) with consideration given to patient literacy, economic status, language, and cultural norms

	• Self-management skills and goals are routinely outlined and monitored
**6. Interdisciplinary Teamwork**	
6.1 Care Team	• Specialty healthcare providers, administrative staff, Veteran patient, family and/or caregiver (when appropriate) work together towards a shared treatment goal
6.2 Sharing of Treatment Information, Case Review, Care Plans, and Feedback	• Regular meetings to discuss complex cases with routine sharing of information and care plans supported by an organizational culture of open communication and collaboration<br>• Routine integrated care team processes, such as huddles and team meetings<br>• Provides feedback (verbal and/or written) to team members on the same day as referral, and as indicated during follow-up services<br>• Utilization of individual and/or group Shared Medical Appointments
6.3 Integrated Care Team Training and Competency Development	• Training events regularly include all staff and providers<br>• Learning materials and interactions reinforce and target areas for improvement emphasizing integrated teamwork
**7. Systematic Quality Improvement (QI)**	
7.1 Use of Quality Metrics for Improvement and Reporting	• Monthly monitoring of access-related metrics<br>• Routine incorporation of measurement with ongoing monitoring of key performance metrics<br>• Responds to findings using formal improvement strategies (e.g., needs assessment, Plan Do Study Act cycles) and routine implementation of improvement projects with demonstration of progress<br>• Regular review (at least annually) of program vision, mission, and goals<br>• Encourage and support team-based quality improvement work
**8. Sustainment**	
8.1 Individual Well-Being, Resilience, and Retention	• Provider labor mapping is regularly reviewed<br>• Individualized performance targets align with the provider's specific roles and duties in the specialty medicine clinic(s), including engagement in non-clinical critical activities<br>• Individualized performance targets are reviewed and discussed regularly, and adjusted as needed based on the specialty medicine setting and provider-specific responsibilities<br>• Staff have knowledge of and access to well-being resources (e.g., EAP, Stress First Aid)<br>• Staff are encouraged to ask for help as needed<br>• Offer available HR policies and flexibilities (e.g., flexible work schedules, telework)<br>• Active and regular recognition of contributions

8.2 Team Well-being and Resilience	• Daily huddles and/or team meetings frequently incorporate a Whole Health focus or voluntary Whole Health activity<br>• Meetings are time-limited to allow space between scheduled events (e.g., limit to 25 or 50min)
8.3 Program Sustainability	• Staff are provided with resources and training on best practices for documentation and coding with specific attention to integrated care; Ongoing training is recommended<br>• Services are available via multiple modalities of care (in-person, video, telephone), with all modalities offered to all Veteran patients based on preference and clinical judgment
